# The Accuracy of Evaluation of the Requirements of the Standards IEC 61000-3-2(12) with the Application of the Wideband Current Transducer

**DOI:** 10.3390/s24113693

**Published:** 2024-06-06

**Authors:** Ernest Stano, Slawomir Wiak

**Affiliations:** Institute of Mechatronics and Information Systems, Lodz University of Technology, 90-537 Lodz, Poland; slawomir.wiak@p.lodz.pl

**Keywords:** current transducer, conversion accuracy, IEC 61000-3-2, IEC 61000-3-12, electromagnetic compatibility, current harmonics

## Abstract

The aim of this paper is to determine the conversion accuracy of the Danisense DC200IF (Danisense A/S, Taastrup, Denmark) wideband current transducer for its possible application to test electromagnetic compatibility requirements of the standards IEC 61000-3-2 and IEC 61000-3-12 with the digital power meter Yokogawa WT5000 (Yokogawa Electric Corporation, Tokyo, Japan). To obtain this goal for distorted current of main frequency equal to 50 Hz and in the frequencies range of higher harmonics from 100 Hz to 2500 Hz its amplitude error and phase shift are evaluated. Moreover, the measurable level of higher harmonics with the rated accuracy of the used precision power analyzer is also investigated. Finally, the measuring system is applied to determine the RMS values of current harmonics produced by the audio power amplifier in order to assess its compliance with the standard IEC 61000-3-12.

## 1. Introduction

The assessment of current harmonics generated by equipment connected to the power system is of paramount importance in the field of electromagnetic compatibility (EMC). Efficient assessment serves as a preventive measure against the propagation of electromagnetic disturbances and safeguards the overall power quality [1,2,3,4,5,6]. The standards IEC 61000-3-2 and IEC 61000-3-12 define the limits for the harmonic current generated by the low voltage equipment connected to the power system [7,8]. The standard IEC 61000-3-12 specifically addresses scenarios where the rated current RMS value of the device under test exceeds 16 A and extends up to but does not exceed 75 A. Meanwhile, the standard IEC 61000-3-2 comes into play when the rated current RMS value of the device under test does not exceed 16 A per phase. These standards serve as foundational benchmarks for ensuring the compliance of electrical and electronic devices with the stipulated harmonic current limits. Wideband current transducers play a key role in extending the measurement capabilities of test equipment or acting as a current-to-voltage converter. The wideband current transducer tested and considered in this paper has the potential to extend the current measurement range of the associated equipment or to act as a current-to-voltage converter, especially when coupled with an output resistance of, for example, 10 Ω. Another solution is to use conventional inductive current transformers, which are capable of transforming a distorted current with sufficient accuracy [9,10]. However, their metrological properties can be affected by the self-generation phenomenon caused by the non-linearity of the magnetization characteristic of the magnetic core [11]. This behavior is not observed with electronically controlled current transformers [12,13,14,15,16].

The determination of the harmonic current error and phase angle values of the tested current transducers during the transformation distortion current requires the use of measurement systems which may be supplied by a high current source [1,17]. Another solution is to use the ampere-turns method, where the tested current transducer is equipped with an additional primary winding with a known number of turns [18]. This approach eliminates the need to use the high current system and supplies the tested current transducer with a reduced value of the distorted current. This method can only be used with window type current transducers. Other approaches are presented in the papers [19,20,21,22]. The authors in reference [20] presented a measurement system using a portable digital acquisition system together with low-cost voltage and current acquisition cards. In addition, the on-line calibration of electronic current transformers was proposed in another reference [22]. The tests carried out showed a simplification of the calibration procedure, leading to a reduction in costs. The use of appropriate instrument transformers is essential for the correct measurement of power quality and can help to identify sources of disturbance propagation and increase the reliability of the power system [23,24,25].

The aim of this research is to evaluate the conversion accuracy of the Danisense DC200IF (Danisense A/S, Taastrup, Denmark) wideband current transducer. The focus is on its potential application for electromagnetic compatibility testing according to the IEC 61000-3-2 and IEC 61000-3-12 standards. The chosen instrument for this evaluation is the Yokogawa WT5000 (Yokogawa Electric Corporation, Tokyo, Japan) digital power meter [26]. In order to achieve this objective, the laboratory study of the accuracy of the Danisense DC200IF (Danisense A/S, Taastrup, Denmark) transducer during transformation distorted current was investigated. Specifically, the analysis covers the main frequency of 50 Hz and extends to higher harmonics within the frequency range of 100 Hz to 2500 Hz. Two key parameters, amplitude error and phase shift, are evaluated to measure the accuracy of the transducer in measuring distorted currents. In addition, a precision power analyzer is used to investigate the measurable level of higher harmonics within this frequency range to ensure that the transducer is matched to the instrument’s rated accuracy. Therefore, the accuracy of the measured RMS values of harmonics by the Yokogawa WT5000 (Yokogawa Electric Corporation, Tokyo, Japan) is several orders of magnitude greater than that of the transducer under test, so its accuracy does not affect the results obtained. In the final stage of the study, the developed measurement system is practically applied to determine the RMS values of the current harmonics generated by an audio power amplifier. This approach is used to assess the compliance of the audio power amplifier with the IEC 61000-3-12 standard, which specifies electromagnetic compatibility requirements. The overall objective is to provide a comprehensive evaluation of the performance of the Danisense DC200IF (Danisense A/S, Taastrup, Denmark) transducer and its potential suitability for performing tests related to the electromagnetic compatibility requirements specified in the standards.

## 2. The Standards IEC 61000-3-2 and IEC 61000-3-12

The part 3–12 gives the limits for the emission of current harmonics by the equipment with a rated current above 16 A and up to and including 75 A connected to the public low-voltage power supply system (i.e., voltage not exceeding 1000 V AC and 1500 V DC) where a rated frequency of 50 Hz or 60 Hz is specified.

The limits are expressed in terms of the permissible harmonic parameters *THC* and *PWHC*, as well as by the maximum permissible percentage values of the individual current harmonics.

(1) $$THC=\sqrt{\sum_{h=2}^{40}I_{h}^{2}},$$

(2) $$PWHC=\sqrt{\sum_{h=14}^{40}{h\cdot I}_{h}^{2}},$$

The standard IEC 61000-3-2 specifies the limits for harmonic current emissions from electrical and electronic equipment with an input current rating of up to and including 16 A per phase.

The limits are expressed in terms of the RMS values of the individual current harmonics.

This paper evaluates the accuracy of current harmonic conversion using the wideband current transducer. The analysis performed concerns the determination of its potential impact on the results of the assessment of current harmonics in low-voltage networks when the connected equipment complies with the standards IEC 61000-3-2 and IEC 61000-3-12 [7,8].

## 3. The Objects of the Research and the Measuring Setup

The objective of this study was to evaluate the measurement accuracy of the Danisense DC200IF (Danisense A/S, Taastrup, Denmark) wideband current transducer in meeting the requirements of the IEC 61000-3-2 and IEC 61000-3-12 standards. The focus was on assessing the amplitude error and phase shift for distorted current with a primary frequency of 50 Hz, extending to higher harmonics in the range of 100 Hz to 2500 Hz. The Yokogawa WT5000 (Yokogawa Electric Corporation, Tokyo, Japan) digital power meter was used as the primary measurement instrument, providing accurate measurements of both current amplitude and phase. The technology used was Fluxgate with closed-loop compensation, which had a fixed excitation frequency and second harmonic zero flux detection, which provided greater accuracy and stability [27]. Such devices have a wide range of applications, including power electronics testing, renewable energy systems, power quality monitoring, and high-precision current measurement in various industrial processes [28,29,30]. They are also used in the testing and development of electric vehicles (EVs) and hybrid electric vehicles (HEVs), as well as in the aerospace and defense industries. They also offer high accuracy, low noise, and a wide bandwidth, making them suitable for a variety of current sensing applications. Declared in the product specification is a measurement bandwidth with an amplitude error and phase shift not exceeding ±0.1%/° from DC to 5000 Hz. However, the devices’ operating bandwidth is up to 200 kHz with a reduced conversion accuracy of ±10% and ±2°, respectively.

**Figure 1:**
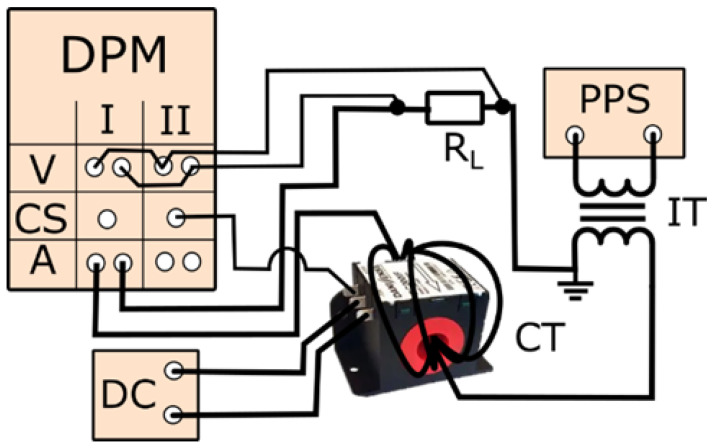
The measuring setup for evaluating the accuracy of tested current transducer. In Figure 1 the following notations are used: DPM is digital power meter/analyzer (V is voltage terminals, CS is current sense terminal, and A is current terminals), CT is current transducer, DC is DC power supply for CT, PPS is programmable power source, R_L_ is load resistor of the PPS, and IT is insulation transformer.

The measuring setup for evaluating the wideband current transducer conversion accuracy of distorted current harmonics is presented in Figure 1.

The experiment was carried out in a controlled laboratory environment with the current transducer connected to the Yokogawa WT5000 (Yokogawa Electric Corporation, Tokyo, Japan) power meter. A programmable power source was used to generate the distorted currents, which was capable of producing a range of harmonic components at different amplitudes and phase angles relative to the fundamental frequency. This setup ensured the ability to simulate different types of electrical noise commonly encountered in industrial applications, which provided a robust test environment for the transducer. The tests of the current transducer were performed in the rated ampere-turns conditions. The RMS values of current in the single wire were equal to 2.5 A, 5 A, 7.5 A, and 10 A. Therefore, the RMS values of the equivalent currents for 20 wires passed through the window of tested transducer were equal to 50 A, 100 A, 150 A, and 200 A which covered the entire range of the transducer under test. The programmable power source was used to supply the measuring system with the distorted current to ensure adjustable RMS values of harmonics and their phase angle in relation to the main component. The evaluation of the Danisense DC200IF (Danisense A/S, Taastrup, Denmark) current transducer was structured into three distinct stages, each designed to systematically test the transducer’s accuracy under different conditions, reflecting realistic electrical disturbances. In the initial stage of the tests, the primary current waveform was carefully shaped to replicate the permissible single harmonic current for conditions with an *R_SCE_* equal to 33, as outlined in Table 1. This setup was chosen to mimic a specific power system scenario where the harmonic current generation is limited by the power system’s capacity to absorb disturbances without significant voltage distortion. The phase angle for all higher harmonics was uniformly set to 0 degrees to simplify the initial analysis and focus solely on amplitude accuracy. In addition, the percentage values of odd higher harmonics above the 13th order were deliberately set to a minimum value of 0.5% to test the transducer’s sensitivity and accuracy in detecting lower harmonics, which are critical in certain applications such as audio equipment and sensitive measurement instruments. The second stage involved configuring the primary current waveform to emulate conditions based on the current emission limits for equipment classified under Class A, in accordance with Table 2. This stage was particularly crucial for assessing the transducer’s performance against defined regulatory criteria that are commonly used in commercial and industrial electrical equipment. By simulating these specific emission limits, the study aimed to validate whether the transducer could accurately measure and thus ensure compliance with established EMC standards. The final stage of the laboratory studies focused on accuracy testing of distorted currents containing multiple higher harmonics, with the percentage of each harmonic set at 5% of the main component at a frequency of 50 Hz. This test condition was designed to replicate more complex and dynamic real-world scenarios, where multiple types of equipment and electronic devices generate a diverse range of harmonics. Such conditions are typical in industrial environments with a mix of heavy machinery and modern electronic equipment, thereby testing the transducer’s capability to accurately measure in an environment with varied harmonic disturbances. Through these three stages, the test methodology assessed not only basic functionality and compliance with standards, but also the robustness and reliability of the transducer under complex, real-world operating conditions. This structured approach ensures a thorough evaluation of the transducer’s performance across a spectrum of possible scenarios, providing comprehensive insight into its practical applications and limitations.

## 4. Results

To characterize the transformation accuracy of the current transducer the amplitude error for the hk harmonic of the distorted current is defined by the following equation:

(3) $$∆I_{m}=\frac{{k_{CT}\cdot I}_{hCT}-I_{hPA}\cdot z}{I_{hPA}}\cdot 100\%,$$

where *k_CT_* is the current ratio of the tested current transducer 1000 A/A, *IhCT* is the RMS value of the current higher harmonic measured by the digital power analyzer with the current transducer, *I_hPA_* is the RMS value of the current higher harmonic measured directly by the digital power analyzer, and *z* is the number of primary current test coil turns.

The values of phase shift specified for the hk harmonic of the distorted current may be determined from the following equation:

(4) $${\delta \varphi }_{hk}=\varphi_{hCT}-\varphi_{hPA},$$

where φ_hCT_ is the phase angle between hk and the fundamental harmonics of the distorted secondary current measured by the digital power analyzer with current transducer, and φ_hPA_ is the phase angle between hk and the fundamental harmonics of the distorted current measured directly by the digital power analyzer.

**Figure 2:**
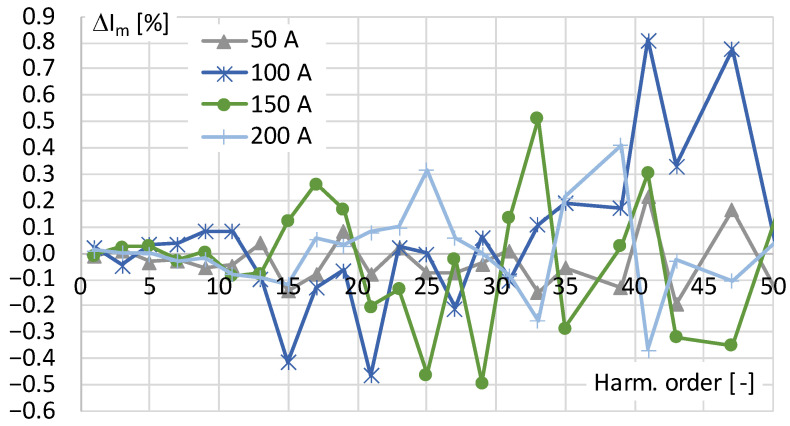
The amplitude errors of transducer determined for distorted current harmonics presented in Table 1.

The values of the amplitude errors determined for each harmonic of distorted current (Table 1) converted by the current transducer are presented in Figure 2.

The above results indicate that the tested current transducer is characterized by an amplitude error that does not exceed ±1% for higher harmonics of order up to 50th. The increase in its determined value in the frequency range above 450 Hz is caused by the fact that the RMS values of the distorted primary current harmonic are too low to ensure the nominal measurement accuracy of the precision power analyzer used.

**Figure 3:**
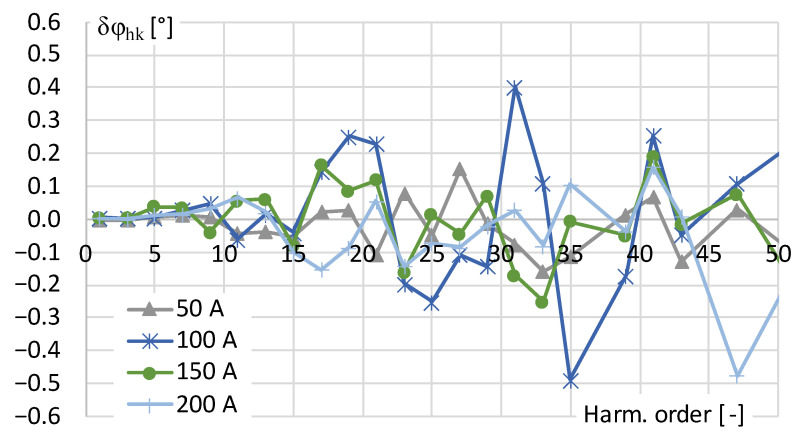
The phase shift of transducer determined for distorted current harmonics presented in Table 1.

The values of phase shift determined for each harmonic of distorted current are presented in Figure 3.

The results in Figure 3 show that the phase shift values for all the higher harmonics in the frequency range tested are less than ±0.6°. However, because the percentage values of odd higher harmonics above 13th order were set to 0.5% the determined conversion accuracy decreases. This is caused by the fact that the measured higher harmonic RMS value is below 1% of the rated current range of the digital power meter and its measuring accuracy is deteriorated. Therefore, the conversion accuracy of the tested current transducer may not be correctly determined.

In the case of the distorted current with harmonics content as presented in Table 2, the conversion accuracy of the tested transducer was evaluated with the RMS value of the main component of the distorted current equal to 5 A. The measured higher harmonic RMS value is slightly below 1% of the rated current range of the digital power meter. Therefore, the measured RMS value of the higher harmonics is only slightly less than 1% of the rated current range of the digital power meter for the 40th higher harmonic. Under these conditions, the amplitude error and phase shift values determined do not exceed ±0.1%/°.

**Figure 4:**
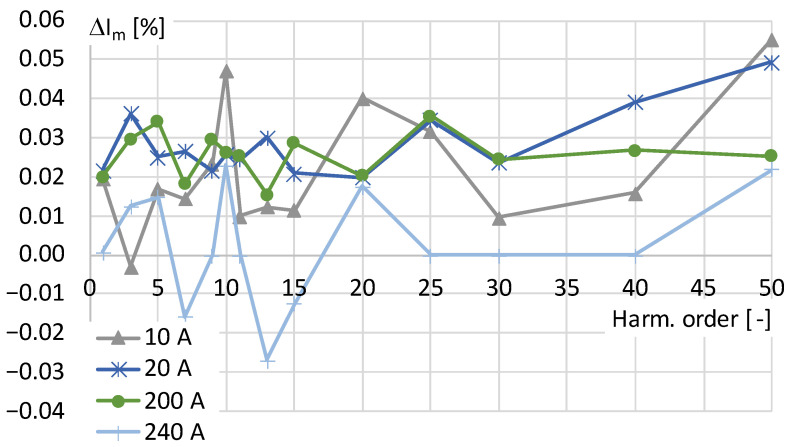
The values of amplitude errors of transducer determined for distorted current with each harmonic equal to 5% of the main component of frequency 50 Hz.

The values of amplitude error determined for conversion of the distorted current harmonics by the tested current transducer in the third considered case are presented in Figure 4.

The above results show that the tested current transducer is characterized by values of amplitude errors that do not exceed ±0.06% for higher harmonics of order up to 50th. The visible fluctuations of the measured values are caused by the measurement uncertainty of the digital power meter used when comparing the results between two channels.

**Figure 5:**
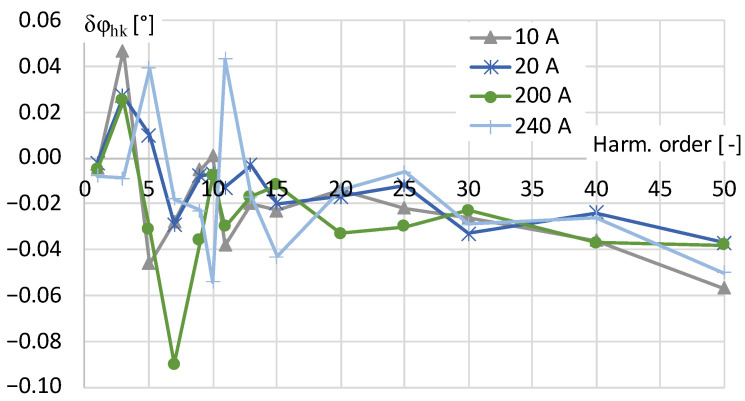
The values of phase shift of transducer determined for distorted current with each harmonic equal to 5% of the main component of frequency 50 Hz.

The phase shift values determined for each harmonic of the distorted current converted by the tested current transducer in the third analyzed case are presented in Figure 5.

The results from Figure 5 show that the phase shift values are less than ±0.1° for all higher harmonics in the frequency range tested. The accuracy of the current transducer tested is in accordance with the value declared by the manufacturer.

## 5. Application

**Figure 6:**
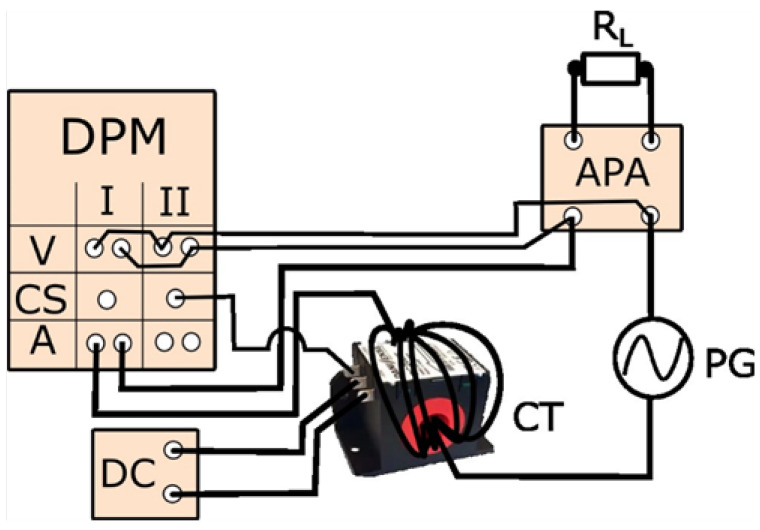
The measuring system for evaluation of the RMS values of harmonics current produced by the audio power amplifier. In Figure 6, the same abbreviation is used as in Figure 1; in addition, APA is audio power amplifier, R_L_ is load of the APA, and PG is power grid.

The tested current transducer is used to determine the current harmonics generated by the audio power amplifier in accordance with the standard IEC 61000-3-12. This unit is characterized by the rated input current 32 A and the rated output active power 5 kW. Figure 6 shows the measurement system used to evaluate the RMS values of the current harmonics generated by the audio power amplifier.

**Figure 7:**
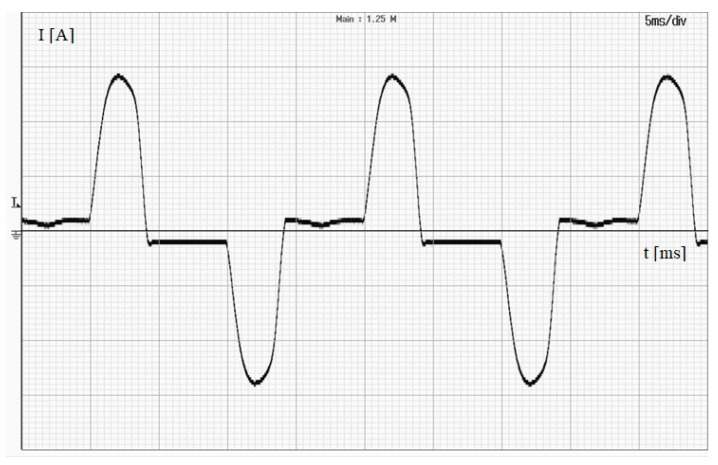
The waveform of the distorted current recorded during emission test of the power amplifier.

The wire with the measured current was pulled through the window of used current transducer six times in order to ensure rated operation conditions. The tests were performed for five different load conditions of the power amplifier. Figure 7 shows the waveform of the distorted current recorded during the emission test of the power amplifier.

The waveform shown in Figure 7 is characteristic of the rectification system used in the power amplifier to supply the DC rail. Therefore, the EMI filter is not used by the manufacturer.

**Figure 8:**
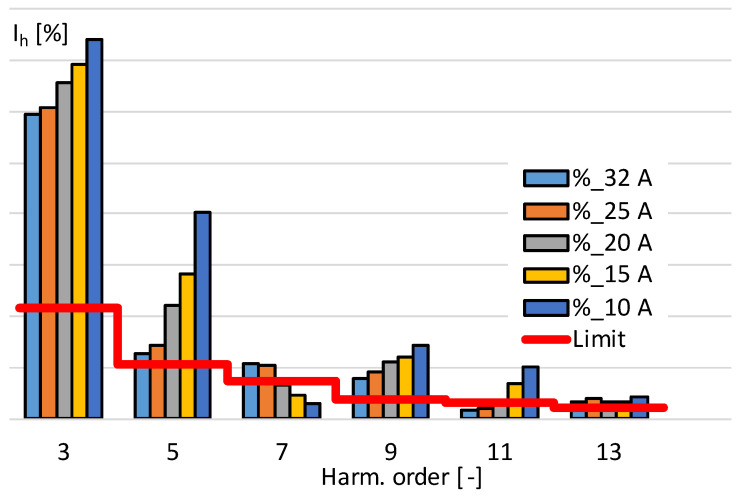
The percentage level of harmonic current emission of the tested power amplifier.

Figure 8 shows the percentage level of harmonic current emission of the tested power amplifier for five different load conditions.

The current drawn by the power amplifier is proportional to the output power. Therefore, for a given output voltage, reducing the load resistance will increase the output current and the required supply current. The emission of current harmonics by the power amplifier exceeds the limits defined in the standard IEC 61000-3-12. The results presented in Figure 8 show that, with the increase in the input current, the percentage value of current harmonics of orders third, fifth, ninth, and eleventh increased.

## 6. Conclusions

The wideband current transducer underwent a comprehensive evaluation focusing on its conversion accuracy, particularly in the area of distorted current higher harmonics. The evaluation covered a frequency range from 50 Hz to 2500 Hz. The results showed that the transducer consistently met the stringent criteria set by the manufacturer. Within this frequency range, the transducer exhibited amplitude error and phase shift that did not exceed ±0.1%/°, as specified by the manufacturer. The accuracy level designated in the laboratory studies means that the transducer can reliably and accurately detect distorted current signals with minimal deviation, enhancing its effectiveness in various electrical environments characterized by higher harmonics. Adherence to the declared accuracy standard is essential to ensure the reliability of measurements in applications where accurate current information is the most important. Furthermore, the integration of this approved current transducer with a digital power analyzer opens up the possibility of evaluating the RMS values of harmonic currents generated by equipment connected to public low-voltage grids. This evaluation meets the requirements of international standards, in particular IEC 61000-3-2 and IEC 61000-3-12. These standards set benchmarks for the permissible levels of harmonic currents injected into the power system by electrical equipment. However, a notable condition for maintaining rated measurement accuracy is to ensure that the lowest measured harmonic RMS value remains above 1% of the current range used. This criterion highlights the importance of having a baseline level of harmonic content within the measurement range to maintain measurement accuracy. It serves as a practical constraint to ensure that the transducer operates optimally and provides reliable data when evaluating harmonic content according to the specified standards. The wideband current transducer has been rigorously tested to demonstrate its ability to maintain accuracy in the presence of distorted higher harmonics. Its integration with a digital power analyzer aligns with industry standards, making it a valuable tool for assessing harmonic currents in low-voltage systems. The requirement for the lowest measured harmonic RMS value emphasizes the need for a minimum harmonic content to ensure the transducer’s rated measurement accuracy.

## Figures and Tables

**Table 1:** Harmonic current emission limits for balanced three-phase equipment in accordance with the standard IEC 61000-3-12:2011 + AMD1:2021.

Min. R_SCE_	Admissible Individual Harmonic Current I_h_/I_1_ [%]						Admissible Harmonic Parameters [%]	
	I_3_	I_5_	I_7_	I_9_	I_11_	I_13_	*THC/I*_1_	*PWHC/I*_1_
33	21.6	10.7	7.2	3.8	3.1	2	23	23
66	24	13	8	5	4	3	26	26
120	27	15	10	6	5	4	30	30
250	35	20	13	9	8	6	40	40
≥350	41	24	15	12	10	8	47	47

Where: *R_SCE_*—short-circuit ratio (it is proportional to the ratio of the 3-phase short-circuit fault level at the point of common coupling to the rated apparent power of the equipment), *THC*—total harmonic current *PWHC*—partial weighted harmonic current, *I*_1_—main current harmonic RMS value, *I_h_*—higher harmonic current RMS value.

**Table 2:** Harmonic current emission limits for equipment class A in accordance with the standard IEC 61000-3-2:2018 + AMD1:2020.

Harm. Order n	Maximum Permissible Harmonic Current [A]
Odd harmonics	
3	2.30
5	1.14
7	0.77
9	0.40
11	0.33
13	0.21
15 ≤ *n* ≤ 39	$0.15\;\frac{15}{n}$
Even harmonics	
2	1.08
4	0.43
6	0.30
8 ≤ *n* ≤ 40	$0.23\;\frac{8}{n}$

## Data Availability

Data are contained within the article.
